# Dynamic Characteristics of Asphalt Concrete as an Impervious Core in Embankment Dams under Varying Temperatures and Stress States

**DOI:** 10.3390/ma16196529

**Published:** 2023-10-01

**Authors:** Xiaoning Han, Zaiqiang Hu, Liangshu Yu, Yuan Pang, Haicheng She, Longfei Zhang, Xiaoliang Wang, Changjun Qi

**Affiliations:** 1 Institute of Geotechnical Engineering, Xi’an University of Technology, Xi’an 710048, China; hxinian@126.com (X.H.); yliangshu@126.com (L.Y.); pangyuan@xaut.edu.cn (Y.P.); 1190711011@stu.xaut.edu.cn (L.Z.); qicj225@163.com (C.Q.); 2 School of Urban Construction, Yangtze University, Jingzhou 434032, China; shehaicheng@126.com

**Keywords:** dynamic triaxial experiment, hydraulic asphalt concrete, maximum dynamic elastic modulus, damping ratio

## Abstract

To reveal the dynamic characteristics of asphalt core embankment dams (ACEDs), we carried out a dynamic triaxial experiment on hydraulic asphalt concrete (HAC) under different temperatures (*T* = 4 °C, 10 °C, 16 °C, and 22 °C) and stress states (*K_c_ =* 1.0, 1.2, 1.4, and 1.6; *σ_3_* = 0.5, 0.6, 0.7, and 0.8 MPa). The results indicate that HAC’s maximum dynamic elastic modulus increased with decreasing temperature, increasing principal stress ratio, and increasing confining pressure. However, the damping ratio showed the opposite trend. Moreover, in order to study the deformation capacity of HAC, 300 cyclic loads were applied to some specimens. At a temperature of 22 °C, the specimens had a tendency to deform axially, but not significantly. With a decrease in temperature, the axial deformation tendency of the specimen gradually weakened or even disappeared. However, a small number of cracks appeared in the aggregate and between the asphalt and the aggregate of the specimen. In order to quantify the dependence of dynamic parameters on temperature, the temperature influence factor of the maximum dynamic elastic modulus and the temperature sensing factor of the damping ratio were defined. The variation in the temperature influence factor of the maximum dynamic elastic modulus with temperature can be described by a logistic function. The temperature sensing factor of the damping ratio increased with an increasing principal stress ratio and peripheral pressure. Finally, maximum dynamic elastic modulus and damping ratio computational models for the interaction of temperatures and stress states were developed using the normalization method. Upon comparison, the dynamic parameters were observed to be very close to those listed in the literature, which verifies the applicability of the computational models of the maximum dynamic elastic modulus and damping ratio.

## 1. Introduction

Hydraulic asphalt concrete (HAC) is a composite material of bitumen, coarse aggregate, fine aggregate, and filler [1]. Owing to its good impermeability, adaptability to deformation, and self-healing properties, it is widely utilized as the core in embankment dams [2,3]. At the 16th International Dam Conference in 1988, it was pointed out that asphalt core embankment dams (ACEDs) are also a suitable dam type for future super-high dams [4,5]. With the implementation of the “double carbon” goal and rapid development of pumped storage power stations in various regions, HAC, as an essential engineering material, will also undergo more significant development.

Since the 1960s, nearly 200 ACEDs have been constructed in various countries worldwide [6,7]. In engineering applications, ACEDs not only withstand static loads but also endure dynamic loads. Static loads include structural deadweight, water pressure, and the weight of structural attachments and equipment. Dynamic loads, however, incorporate earthquakes, wind loads, and water strikes [8], especially in ACEDs located in an earthquake-prone area. Additionally, the dynamic properties of HAC are related to various factors, including internal factors, such as asphalt content and asphalt mixture gradation, and external factors, such as temperatures, principal stress ratios, and confining pressures. Therefore, in order to investigate the effects of multiple external factors on the dynamic properties of HAC, the subject of this paper (HAC was used as a core in an embankment dam) needs to be at a specific mixing ratio.

The strain rate effect and cyclic loading effect are the two main aspects of studying the dynamic properties of concrete [9]. The same is true for the study of the dynamic properties of HAC. The study on the strain rate effect of HAC is relatively complete. Wang et al. found a significant decrease in modulus with a decreasing strain rate [10]. Wang et al. investigated the behavior of the stress–strain strength of HAC under different temperatures and shear strain rates through shear tests [11]. Ning et al. discovered that temperature and strain rate had a significant impact not only on the stress–strain characteristics of HAC but also on its failure modes using an axial compressive test [3]. Moreover, they also established calculation models that consider the interactions between temperature and strain rate to calculate the compressive strength and elastic modulus [3]. These conclusions were obtained from various mechanical perspectives, and calculation models, which include multiple factors, have been established.

Regarding the investigation of the cyclic loading effect, Yu et al. investigated the effect of asphalt content on the dynamic properties of HAC [12]. Feizi-Khankandi et al. discovered that, by increasing the temperature, the shear modulus decreases, but it increases as the principal stress ratios and confining pressures are increased [13]. Moreover, they further indicated that there was no long-term degradation phenomenon for specimens on which 10,000 cycles were imposed [13]. These test results were also confirmed by Wang et al. [14] and Akhtarpour et al. [15]. In addition, Wang and Hoeg developed an empirical expression to determine the cyclic modulus [14]. Wang et al. proposed a modified dynamic modulus expression based on the results of a dynamic triaxial experiment [16]. However, these studies mainly focused on qualitative descriptions and quantitative analyses of single factors.

For this purpose, the dynamic characteristics of HAC, under a specific mixing ratio, at different temperatures (*T* = 4 °C, 10 °C, 16 °C, and 22 °C) and stress states (*K_c_* = 1.0, 1.2, 1.4, and 1.6; *σ_3_* = 0.5, 0.6, 0.7, and 0.8 MPa) were investigated in this paper, employing a vibration triaxial instrument. The influences of different temperatures and stress states on the maximum dynamic elastic modulus, and damping ratio of HAC, were explored. In addition, the deformation characteristics of HAC at different temperatures and principal stress ratios were investigated via 300 cyclic loading tests. Finally, empirical formulas were proposed for the temperature influence factors of the maximum dynamic elastic modulus and temperature sensitivity factors of the damping ratio. Calculation models for the maximum dynamic elastic modulus and damping ratio of the interaction between temperatures and stress states were established.

## 2. Experimental Section

### 2.1. Mix Design and Specimen Preparation

HAC partially replaces the cement in ordinary concrete with mineral fillers and bitumen as binders. The asphalt was Karamay 70# asphalt. Alkaline rock (limestone) was used as the aggregate. The raw rock was crushed into a coarse aggregate (19–2.36 mm), fine aggregate (2.36–0.075 mm), and filler (<0.075 mm) according to particle size. Using a square-hole sieve, the coarse aggregate was classified into 5 grades, namely, 19–16 mm, 16–13.2 mm, 13.2–9.5 mm, 9.5–4.75 mm, and 4.75–2.36 mm. The fine aggregates were also divided into 5 grades, namely, 2.36–1.18 mm, 1.18–0.6 mm, 0.6–0.3 mm, 0.3–0.15 mm, and 0.15–0.075 mm. The properties of this asphalt are listed in Table 1.

The mix design of HAC used as a core in embankment dams originated from road asphalt concrete (RAC) [18]. In comparisons with RAC, the bitumen, fine aggregate, and filler contents of HAC have been found to be higher than those of RAC [19,20,21,22]. In designs of ACEDs, the bitumen aggregate ratio generally varies from 6.5% to 7.3%, the filler content is less than 15% [18,19], and the graded index varies from 0.36 to 0.42 [21]. Further research has found that, in order to achieve flexibility during earthquake loading, the range of 6.5–8.5% bitumen content is optimal [14,23]. According to the variation range of various factors in the above literature, the selection of the levels of the factors for this study is shown in Table 2.

**Figure 1:**
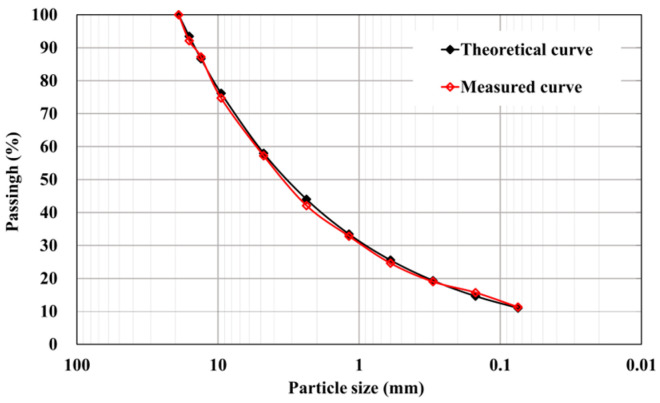
The aggregate gradation curve.

Porosity and splitting tests were conducted on asphalt concrete specimens using orthogonal test methods [24,25]. Furthermore, three mix ratios were initially selected using the extreme difference method. Then, the mechanical properties of the asphalt concrete specimens under each mix ratio were tested separately. The test results were comprehensively evaluated based on the entropy weight TOPSIS method. Finally, the mix ratio with a bitumen aggregate ratio of 6.5%, filler of 11%, and grading index of 0.39 was selected. The aggregate gradation curve is shown in Figure 1, and the asphalt mixture gradation is shown in Table 3.

**Figure 2:**
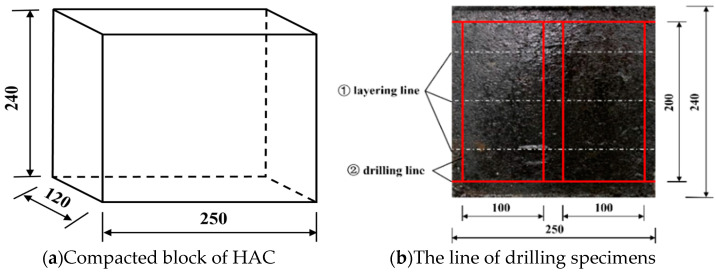
Compacted block of HAC and the line of drilling specimens (unit: mm).

Due to the fact that, when used as the impervious core in embankment dams, HAC is placed and compacted layer by layer, and asphalt blocks were prepared using a compaction method simulating the field compaction to be closer to the core wall sampling in actual engineering [11,26]. Figure 2a shows a compacted block of HAC, while Figure 2b shows how 2 cylindrical specimens were drilled out from the block. The porosity of the specimens was less than 2.0%. The porosity is calculated using the density method, *n* = (1 − *ρ/ρ_max_*) × 100, where n is the porosity of the specimen, *ρ* is the density of the specimen, and ρ_max_ is the maximum density of asphalt mixture proportion. ρ_max_ is 2.470 g/cm^3^ in this experiment.

### 2.2. Experimental Setup and Procedure

The asphalt concrete core is usually positioned in the central region of the embankment dam and protected by the embankment on the sides [8]. As a result, the environmental conditions in that area remain relatively stable. The temperature in the asphalt core in embankment dams during a dam operation is commonly between 5 °C and 20 °C [11]. According to the actual temperature range in which the asphalt concrete core is located [10,11], four temperatures were set, 4 °C, 10 °C, 16 °C, and 22 °C. Considering the varying stress states experienced by the asphalt concrete core during the initiation of dynamic action, four principal stress ratios and four confining pressures were set, namely *K_c_* = 1.0, 1.2, 1.4, and 1.6, and *σ_3_* = 0.5, 0.6, 0.7, and 0.8 MPa. A total of 64 sets of tests were conducted with three specimens set up for each set of tests, and the results were averaged. In addition, to study the deformation characteristics of HAC, 300 cyclic loads were applied to the specimen under the same test conditions. There were 64 groups, with 1 group containing 1 specimen.

**Figure 3:**
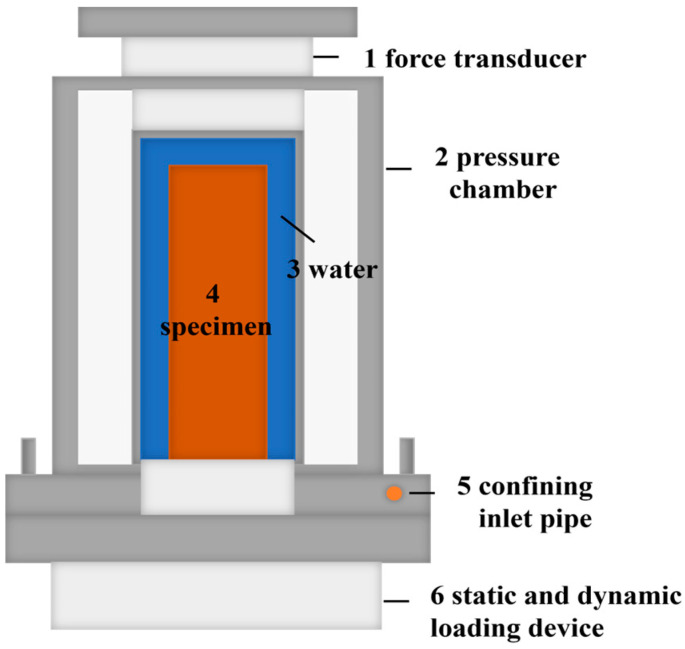
The vibration triaxial instrument.

Figure 3 shows the vibration triaxial instrument. The instrument mainly consists of a pressure chamber, static stress application system, dynamic stress application system, and measurement system. The confining pressures are applied via the pneumatic system, the static or dynamic force is applied through the hydraulic system, and the test data are collected through a computer.

**Figure 4:**
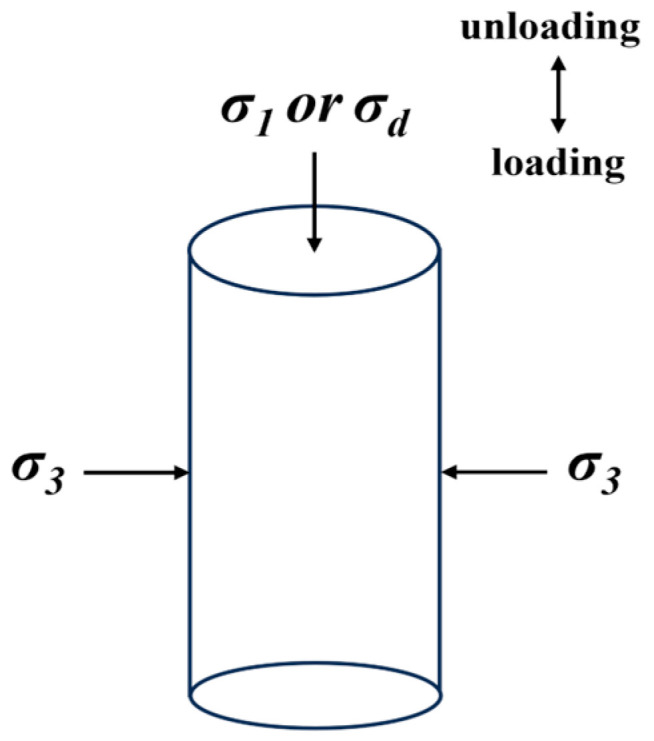
The force form of the specimen.

The instrument was installed in the constant-temperature chamber to minimize the impact of the change in the temperature of the specimens during the test on the test results. The specimens were placed in it for 24 h at the setting temperature [27]. According to the testing specifications of HAC [17], the specimens were tested after consolidation for 0.5 h under the specified principal stresses (*K_c_ = σ_1_/σ_3_*). A certain amount of lateral pressure *σ_3_* and axial *σ_1_* pressure was applied to the specimen. The applied pressures *σ_1_* and *σ_3_* were controlled by readings from standard pressure gauges. Finally, dynamic stresses *σ_d_* were applied to the specimens after they completed consolidation. Dynamic stresses of increasing magnitude were applied to the specimen in stages. The dynamic stress was vibrated 5 times per stage at a frequency of 1 Hz. The force form of the specimen is shown in Figure 4.

## 3. Experimental Results and Analysis

### 3.1. Maximum Dynamic Elastic Modulus

**Figure 5:**
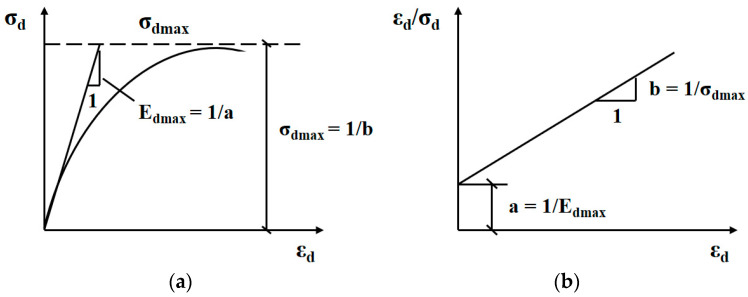
Hyperbola of dynamic stress–dynamic strain relationship. (**a**) The hyperbolic curve of *σ_d_-ε_d_*. (**b**) Transforming the hyperbola of vertical coordinates.

The maximum dynamic elastic modulus reflects the ability of HAC to resist dynamic loads and is an important parameter in the dynamic analysis of HAC. According to the actual test results, the dynamic stress and dynamic strain relationship can be approximated as a hyperbolic relationship. The maximum dynamic elastic modulus is the initial tangent modulus. It is shown in Figure 5. The calculation formula is shown in Equations (1) and (2) [28].

(1) $$\sigma_{d}=\frac{\varepsilon_{d}}{a+b\varepsilon_{d}}$$

(2) $$\frac{1}{E_{d}}=\frac{\varepsilon_{d}}{\sigma_{d}}=a+b\varepsilon_{d}$$

Here, *σ_d_* is the dynamic stress; MPa *ε_d_* is the dynamic strain; *E_d_* is the dynamic elastic modulus, MPa is *a* = 1/*E_dmax_*; and *b* = 1/*σ_dmax_*.

**Figure 6:**
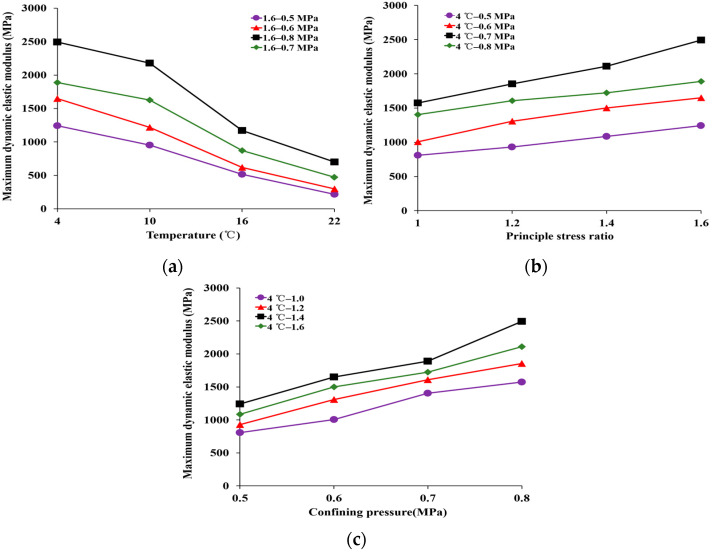
Maximum dynamic elastic modulus of the specimens under different conditions. (**a**) Different temperatures. (**b**) Different principal stress ratios. (**c**) Different pressure.

The maximum dynamic elastic modulus of HAC under various temperatures and stress states is presented in Figure 6.

Based on Figure 6a, when the principal stress ratio was 1.6 and the confining pressure was 0.5 MPa, the maximum dynamic elastic modulus decreased from 1242.28 MPa to 216.60 MP as the temperature increased from 4 °C to 22 °C. The results show that the maximum dynamic elastic modulus of HAC decreased with increasing temperature. This is because the asphalt is the main factor affecting the change in the HAC mechanical properties with temperature. As the temperature rises, the spacing between the asphalt molecules expands, while the adhesion between the molecules diminishes [29].

As shown in Figure 6b,c, with an increase in the principal stress ratio from 1.0 to 1.6, the maximum dynamic elastic modulus increased from 808.50 to 1242.28 MPa at *σ_3_* = 0.5 MPa and *T* = 4 °C, and it increased from 1574.98 to 2492.45 MPa at *σ_3_* = 0.8 MPa and *T* = 4 °C. The results show that the maximum dynamic elastic modulus increases with an increase in the principal stress ratio and confining pressure. This is due to the fact that the increase in the principal stress ratio and confining pressure has a stiffening effect on the specimen as a whole. Moreover, the greater the confining pressure, the greater the lateral pressure received by the specimen [30]. The specimen was under lateral compression. So, the maximum dynamic elastic modulus increased with the increase in the principal stress ratio and confining pressure. At the same time, with an increase in the confining pressure from 0.5 MPa to 0.8 MPa, the maximum dynamic elastic modulus increased from 808.50 to 1574.98 MPa at *K_c_* = 1.0 and *T* = 4 °C, and it increased from 1242.28 to 2492.45 MPa at *K_c_* = 1.6 and *T* = 4 °C. The results show that the bigger the principal stress ratio and confining pressure, the higher the temperature sensitivity of the maximum dynamic elastic modulus.

Moreover, these data also demonstrate that the temperature exerts a significant influence on the maximum dynamic elastic modulus of HAC. For example, the magnitude of change in maximum dynamic elastic modulus due to the temperature, confining pressure, and principal stress ratio are 82.56%, 69.88%, and 37.77%, respectively, when other conditions are constant.

### 3.2. Damping Ratio

**Figure 7:**
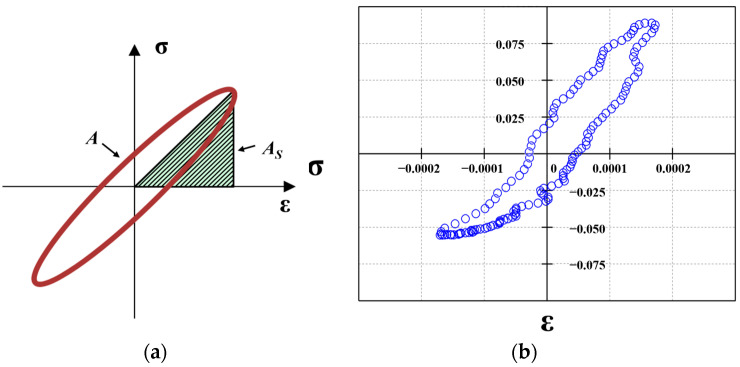
Theoretical and experimental hysteresis loops. (**a**) Theoretical stress–strain hysteresis loops. (**b**) Typical stress–strain hysteresis loops for this dynamic triaxial test.

The damping ratio characterizes the hysteresis of the HAC response to dynamic loads. As shown in Figure 7, the value is calculated based on the hysteresis loop area A of the dynamic stress–strain of HAC and the triangle area, as enclosed by the dynamic stress–strain backbone curve and X-axis. The formula is shown in Equation (3).

(3) $$\lambda =\frac{A}{4\times \pi \times A_{S}}$$

**Figure 8:**
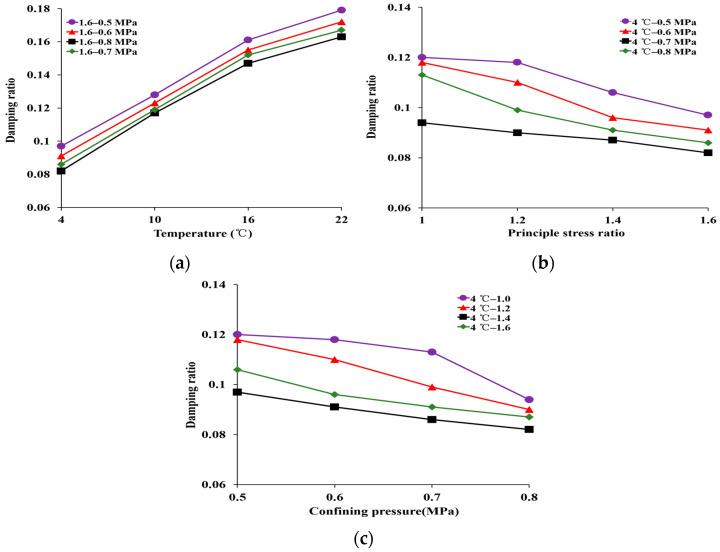
Damping ratio of the specimens under different conditions. (**a**) Different temperatures. (**b**) Different principal stress ratios. (**c**) Different pressure.

The damping ratio of HAC under different temperatures and stress states is illustrated in Figure 8.

As depicted in Figure 8a, with an increasing temperature from 4 °C to 22 °C, the damping ratio increased from 0.082 to 0.163 at *K_c_* = 1.6 and *σ_3_* = 0.8 MPa. This indicates that hysteresis in the response of HAC to dynamic loads is enhanced under high-temperature conditions. This can be attributed to the increase in asphalt viscosity with temperature [31], which creates viscous resistance that impedes the transmission of vibration waves.

As shown in Figure 8b,c, with an increasing principal stress ratio from 1.0 to 1.6, the damping ratio decreased from 0.120 to 0.097 at *σ_3_* = 0.8 MPa and *T* = 4 °C, and it decreased from 0.094 to 0.082 at *σ_3_* = 0.5 MPa and *T* = 4 °C. The results indicate a decrease in the damping ratio with an increase in the principal stress ratio and confining pressure. This is because an increase in the principal stress ratio and confining pressure leads to difficulty in sliding the aggregate in the asphalt matrix. The stability of the specimen is further improved. This also favors the propagation of vibration waves in the specimen. At the same time, with increasing confining pressure from 0.5 MPa to 0.8 MPa, the damping ratio decreased from 0.120 to 0.094 at *K_C_* = 1.6 and *T* = 4 °C, and it decreased from 0.097 to 0.082 at *K_C_* = 1.0 and *T* = 4 °C. It was observed that, as the principal stress ratio and confining pressure increased, the temperature sensitivity of the damping ratio became more pronounced.

Additionally, these data demonstrate that the damping ratio of HAC is most affected by temperature. For example, the magnitude of change in the damping ratio due to temperature, confining pressure, and principal stress ratio are 63.24%, 3.54%, and 20.59%, respectively, when other conditions are constant.

### 3.3. Deformation Characteristics

**Figure 9:**
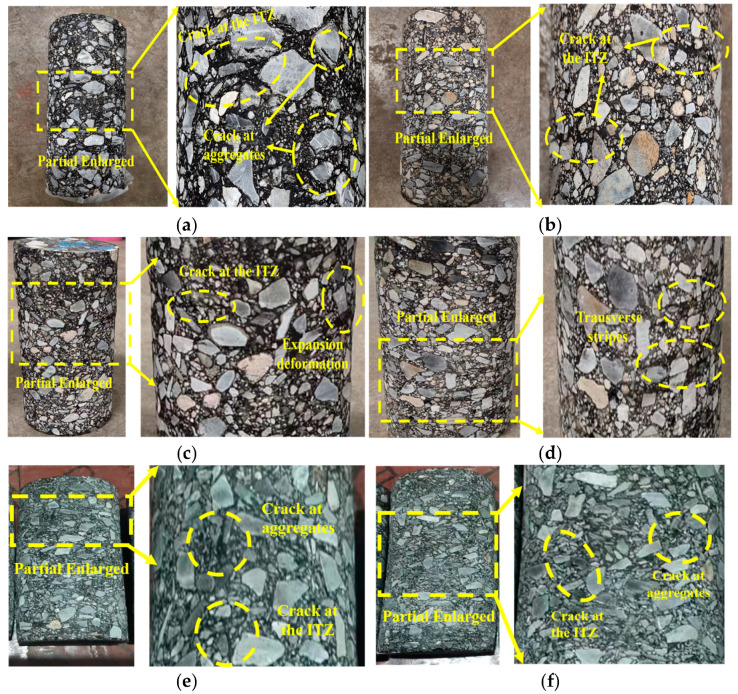
Deformation characteristics of specimens under temperatures and stress states. (**a**) *T* = 4 °C, *K_c_* = 1.6, *σ_3_* = 0.8 MPa. (**b**) *T* = 10 °C, *K_c_* = 1.6, σ_3_ = 0.8 MPa. (**c**) *T* = 16 °C, *K_c_* = 1.6, *σ_3_* = 0.8 MPa. (**d**) *T* = 22 °C, *K_c_* = 1.6, *σ_3_* = 0.8 MPa. (**e**) *T* = 4 °C, *K_c_* = 1.0, *σ_3_* = 0.8 MPa. (**f**) *T* = 4 °C, *K_c_* = 1.6, *σ_3_* = 0.5 MPa.

Figure 9 shows the deformation of the specimens under different temperatures and stress states, subjected to 300 cyclic loads. Permeability tests were carried out on them at the end of cyclic loading, and the permeability coefficients of the specimens all met the [23] requirements. This indicates that HAC has good seismic and impermeable properties.

Figure 9 illustrates that the overall deformation of the specimen was not significant. However, as the temperature increased, the specimen tended to compress axially, accompanied by the appearance of transverse stripes on the free surfaces. This was because, as the temperature increases, the viscosity of asphalt as a bonding material gradually increased [31]. As a result, the residual strain in the specimens increased per cycle during the early stage of cyclic loading. Moreover, this led to an increase in the cumulative permanent deformation of the specimens. In Figure 9a,b, there was not a trend of axial compression on specimens at low temperatures, but a small number of cracks were observed in interfacial transition zones (ITZs) and aggregates. This was due to the difference in strength between the aggregate and asphalt, resulting in the junction layer being the weaker part of the HAC structure [32]. In addition, the bearing capacity of HAC increased as the temperature decreased, eventually causing the aggregate to reach failure strength and crack. Meanwhile, this phenomenon could be enhanced with an increase in the principal stress ratio and confining pressure (Figure 9e,f). The increase in the principal stress ratio and confining pressure favored an increase in the densification of the specimen. Therefore, the specimen load-carrying capacity was increased.

## 4. Computational Models for Maximum Dynamic Elastic Modulus and Damping Ratio

### 4.1. Temperature Influence Factor

**Figure 10:**
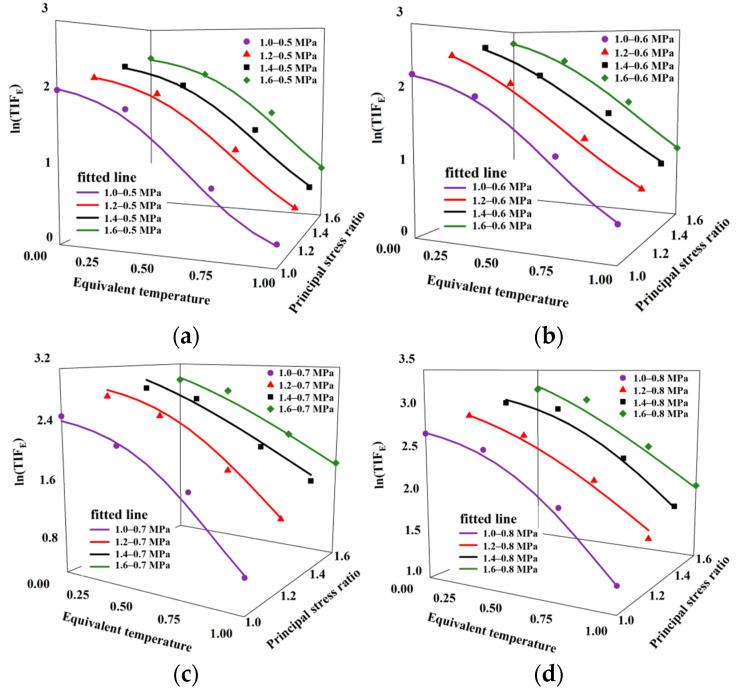
*TIF_E_* of maximum dynamic elastic modulus under different conditions as a function of temperature. (**a**) 0.5 MPa. (**b**) 0.6 MPa. (**c**) 0.7 MPa. (**d**) 0.8 MPa.

To further elucidate the pattern of change in the maximum dynamic elastic modulus within the temperature range of 4–22 °C, we introduced the temperature influence factor (*TIF*) [33] for the maximum dynamic elastic modulus. *TIF_E_* was defined as the ratio of the maximum dynamic elastic modulus at any temperature to that at 25 °C when the stress state remained constant. This is represented by Equation (4):

(4) $${TIF}_{E}=\frac{E_{T}}{E_{25℃}}$$

where $E_{T}$ is the maximum dynamic elastic modulus at 4 °C, 10 °C, 16 °C, or 22 °C, and $E_{25℃}$ is the maximum dynamic elastic modulus at 25 °C. As illustrated in Figure 10, the relationship between ln (*TIF_E_*) and temperature follows a pattern similar to that of an anti-“S” curve under the same stress state. Therefore, the logistic function can be used to describe the variation in the law of the *TIF_E_* with temperature, and this is expressed in Equation (5) [34]:

(5) $$y=\ln\left({TIF}_{E}\right)=\frac{k}{1+aexp(-bT^{*})}$$

where *a*, *b*, and *k* are material parameters that indicate the fitting between the experimental data and formula, and $T^{*}$ is the equivalent temperature. $T^{*}$ can be calculated using Equation (6):

(6) $$T^{*}=\frac{T-T_{l}}{T_{h}-T_{l}}$$

where *T_h_* is the highest temperature within the scope of this study (22 °C), and *T_l_* is the lowest temperature within the scope of this study (4 °C). The values of *a*, *b*, *k*, and *R^2^* were calculated using the least squares method in Table 4. 

As shown in Figure 10, *TIF_E_* decreases with increasing temperature at a certain principal stress ratio and confining pressure, and the magnitude of change is different. This is because, when the temperature is greater than 5 °C, HAC mainly exhibits viscoelasticity [3,31], and its viscosity increases with increasing temperature. The higher the temperature, the smaller the maximum dynamic elastic modulus. At 16–22 °C, *TIF_E_* produces less change with temperature change. In contrast, the elasticity of HAC increases with decreasing temperature. At 4–16 °C, *TIF_E_* produces a large difference with temperature change. In conjunction with Table 4 at 4–22 °C, it can be seen that the logistic function (fitted line in Figure 10) can better express the *TIF_E_* change rule to temperature.

### 4.2. Temperature Influence Factor

**Figure 11:**
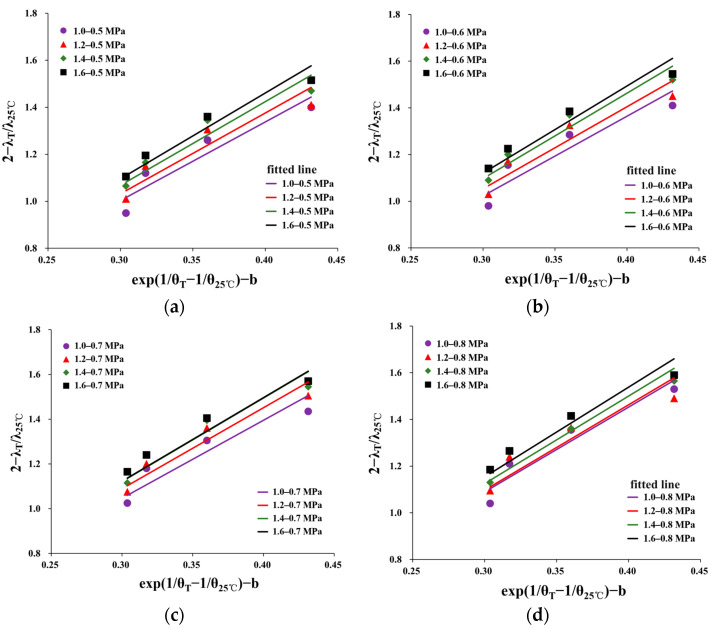
Temperature sensitivity factor of damping ratio under different conditions as a function of temperature. (**a**) 0.5 MPa. (**b**) 0.6 MPa. (**c**) 0.7 MPa. (**d**) 0.8 MPa.

The temperature sensitivity factor [35] was used to further study the changes in the damping ratio with temperature. It was assumed that *B_λ_* is the temperature sensitivity factor of the damping ratio. *B_λ_* was calculated follows:

(7) $$B_{\lambda }=\frac{y}{x}=\frac{\lambda_{T}/\lambda_{25℃}}{\exp(1/\theta_{T}-1/\theta_{25℃})}$$

where *λ_T_* is the damping ratio at *T* °C; *λ_25°C_* is the damping ratio at 25 °C; *θ_T_* is the temperature; and *θ_25°C_* = 25 °C. As the damping ratio decreased with increasing temperature, Equation (7) is a negative correlation. In order to make the value of *B_λ_* directly reflect the sensitivity of the damping ratio of HAC to temperature, Equation (7) can be transformed into a positive correlation through translation and rotation, resulting in the expression shown in Equation (8):

(8) $$B_{\lambda }=\frac{2-y}{x-b}=\frac{2-\lambda_{T}/\lambda_{25℃}}{exp(1/\theta_{T}-1/\theta_{25℃})-b}$$

where *b* is the temperature correction factor (0.7 at 10 °C, 16 °C, and 22 °C or 0.8 at 4 °C); *B_λ_* can be obtained through a regression analysis of the experimental data. Figure 11 depicts (*2* − *λ_T_*/*λ_25°C_*) − (*exp*(*1*/*θ_T_* − *1*/*θ_25°C_*) − *b*) and line fitting. The fitting results are shown in Table 5. 

By observing Figure 11, it is evident that the fitting relationship displays a linear pattern that essentially passes through the origin. Equation (8) effectively describes the temperature-dependent quantitative relationship of the damping ratio across varying principal stress ratios and confining pressures.

Based on the data presented in Table 5, it was observed that the temperature sensitivity factors obtained through fitting showed an increasing trend with higher principal stress ratios and confining pressures. This finding aligned with the previous analysis, confirming that the temperature sensitivity factor effectively can characterize the sensitivity of HAC’s damping ratio to temperature.

### 4.3. Calculation Model for Maximum Dynamic Elastic Modulus and Damping Ratio

To facilitate obtaining the maximum dynamic elastic modulus and damping ratio of HAC under various conditions, it is necessary to mathematically describe the variation in these parameters with respect to various factors.

Relationships between dynamic parameters (the maximum dynamic elastic modulus and damping ratio) and various factors (the temperature, principal stress ratio, and confining pressure) were established through coordinate axes. The maximum dynamic elastic modulus and damping ratio computational models for the interaction of different temperatures and stress states were developed using the normalization method [36,37].

#### 4.3.1. Calculation Model for Maximum Dynamic Elastic Modulus

Based on the maximum dynamic elastic modulus (*E_σ3dmax_*) corresponding to the minimum confining pressure (*σ_3_* = 0.5 MPa) under each principal stress ratio, the *E_dmax_*−*σ_3_* curves can be transformed into *E_dmax_*/*E_σ3dmax_*−*σ_3_* curves, as presented in Figure 9.

**Figure 12:**
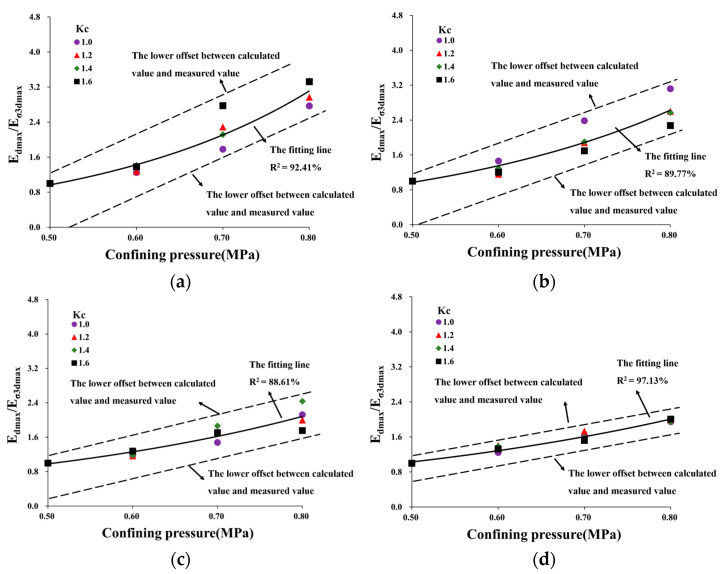
Curves of *E_dmax_/E_σ3dmax_*−σ_3_ at different temperatures. (**a**) 22 °C. (**b**) 16 °C. (**c**) 10 °C. (**d**) 4 °C.

**Figure 13:**
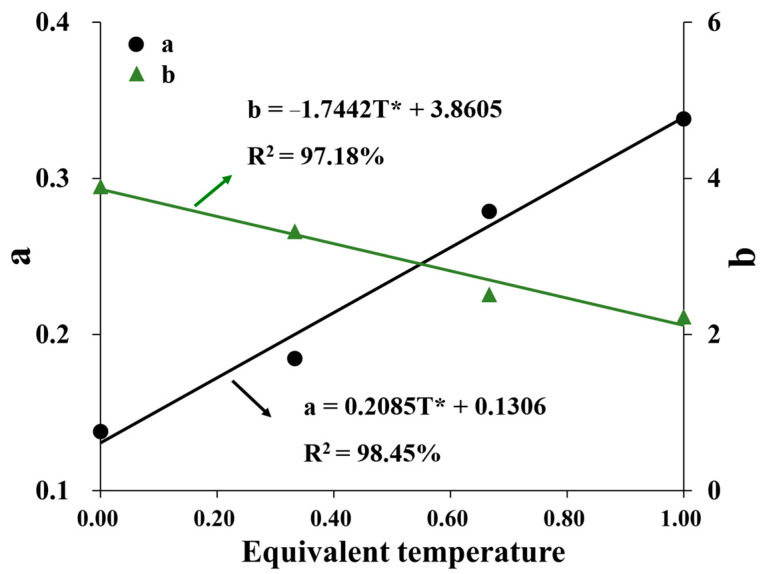
Curves of parameters a and b vs. equivalent temperature.

As shown in Figure 12, the test points of *E_dmax_*/*E_σ3dmax_*−*σ_3_* at different temperatures were distributed within a very narrow area. This indicated that using *E_σ3dmax_* can effectively normalize the *E_dmax_*−*σ_3_* curves at different temperatures, thus allowing for an approximate description by means of a curve. This article proposes directly employing an exponential function to represent the normalized curve in the coordinate system of *E_dmax_*/*E_σ3dmax_*−*σ_3_*. The exponential function can be expressed as follows:

(9) $$\frac{E_{dmax}}{E_{\sigma_{3}dmax}}=aexp(b\sigma_{3})$$

where *a* and *b* are parameters of HAC. The normalized curves at different confining pressures were fitted using Equation (9). The fitting coefficients for all exceeded 0.85. The relationship curves depicting the fitting parameters of *a* and *b* at various temperatures are presented in Figure 13.

According to Figure 13, parameter “*a*” increases approximately linearly, and parameter “*b*” decreases approximately linearly with the increase in the equivalent temperature (Equation (5)), as shown in Equations (10) and (11), respectively.

(10) $$a=0.2085T^{*}+0.1306$$

(11) $$b=-1.7442T^{*}+3.8605$$

**Figure 14:**
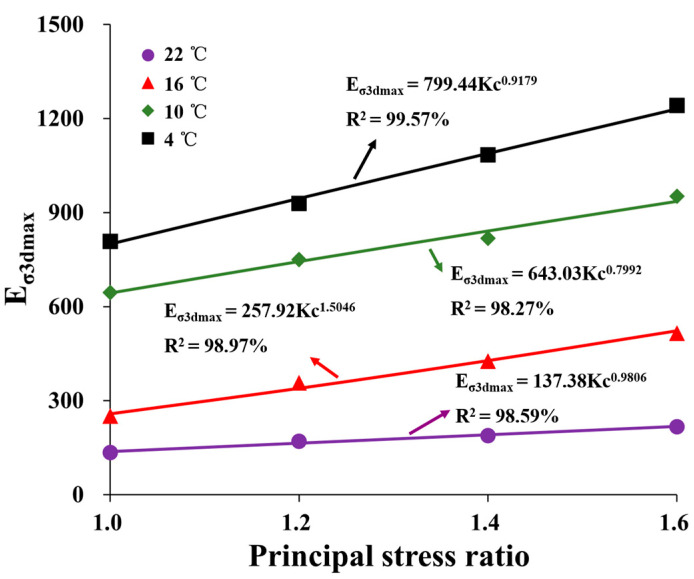
Curves of *E_σ3dmax_*-*K_c_* under different conditions.

**Figure 15:**
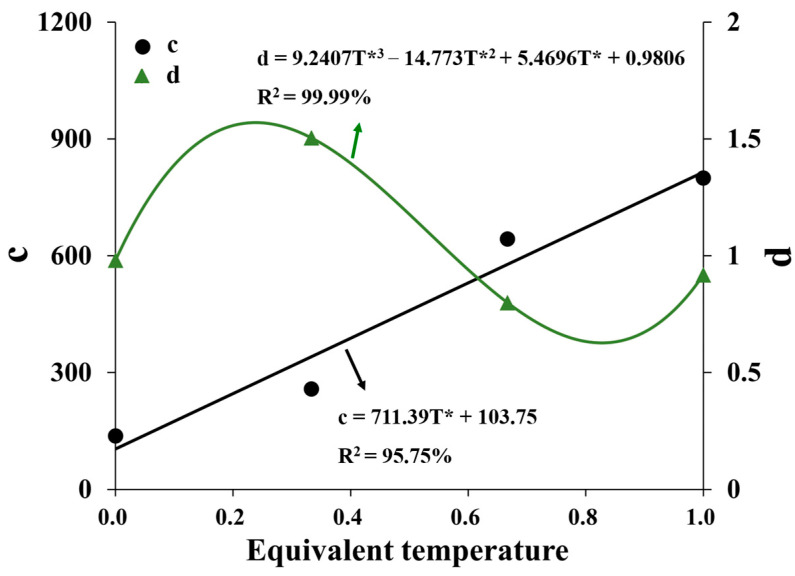
Curves of parameters *c* and *d* vs. equivalent temperature.

Figure 14 shows the test points of *E_σ3dmax_*. The power function can be expressed as follows:

(12) $$E_{\sigma_{3}max}=cK_{c}^{d}$$

where *c* and *d* are parameters of HAC. Equation (12) was used to fit the test points at different principal stress ratios. As shown in Figure 14, the fitting coefficients are all greater than 0.95. The relationship curves of fitting parameters *c* and *d* at different temperatures are shown in Figure 15.

According to Figure 15, the linear and polynomial functions were, respectively, used to fit parameters *c* and *d*, as presented in Equations (13) and (14):

(13) $$c=711.39T^{*}+103.75$$

(14) $$d=9.2407T^{*3}-14.773T^{*2}+5.4696T^{*}+0.9806$$

**Figure 16:**
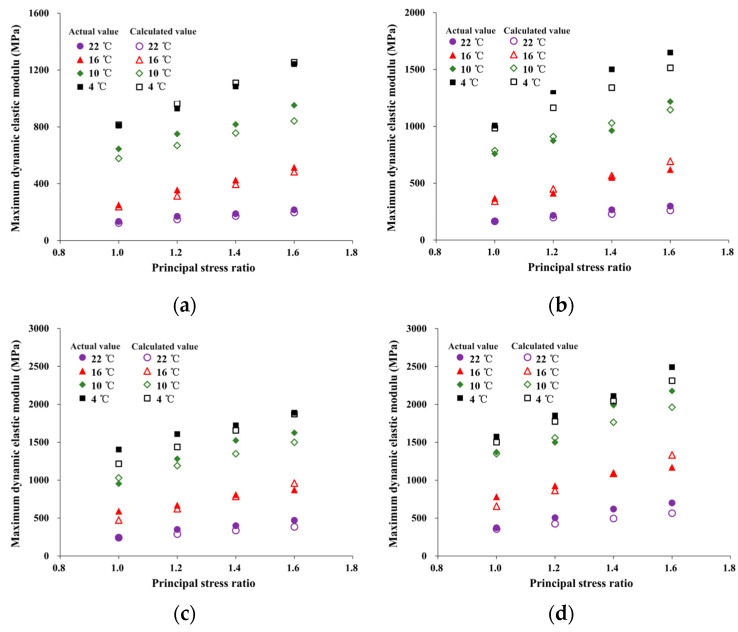
Comparison of the actual and calculated values of maximum dynamic modulus. (**a**) 0.5 MPa. (**b**) 0.6 MPa. (**c**) 0.7 MPa. (**d**) 0.8 MPa.

By combining Equations (9)–(14) and (5), the maximum dynamic elastic modulus normalization curve considering temperatures and stress states can be obtained, as shown in Equation (15):

(15) $$\frac{E_{dmax}}{\left(711.39T^{*}+103.75\right)K_{C}^{\left(9.2407T^{*3}-14.773T^{*2}+5.4696T^{*}+0.9806\right)}\times F_{t}}=\left(0.2085T^{*}+0.1306\right)exp((-1.7442T^{*}+3.8605)\sigma_{3})$$

where *F_t_* is the temperature correction factor (*F_t_* = 1.2 at 22 °C, *F_t_* = 0.7 at 16 °C, and *F_t_* = 1 at 10 °C and 4 °C). Using Equation (15), the maximum dynamic elastic modulus of HAC was calculated under different conditions and compared with experimental results, as shown in Figure 16.

Based on the data in Figure 16, the comparison results demonstrate that the normalization equation proposed in this paper was applicable for reflecting the maximum dynamic elastic modulus of HAC under different temperatures and stress states. The relative error between actual and calculated values was within 20% and mostly within 15%.

Table 6 shows the comparison between the calculation results of Equation (15) and the maximum dynamic elastic modulus provided by [14]. The relative errors are 16.97%, 15.13%, 8.76%, and 5.71%, which are in good agreement with the provided results.

#### 4.3.2. Calculation Model for Damping Ratio

**Figure 17:**
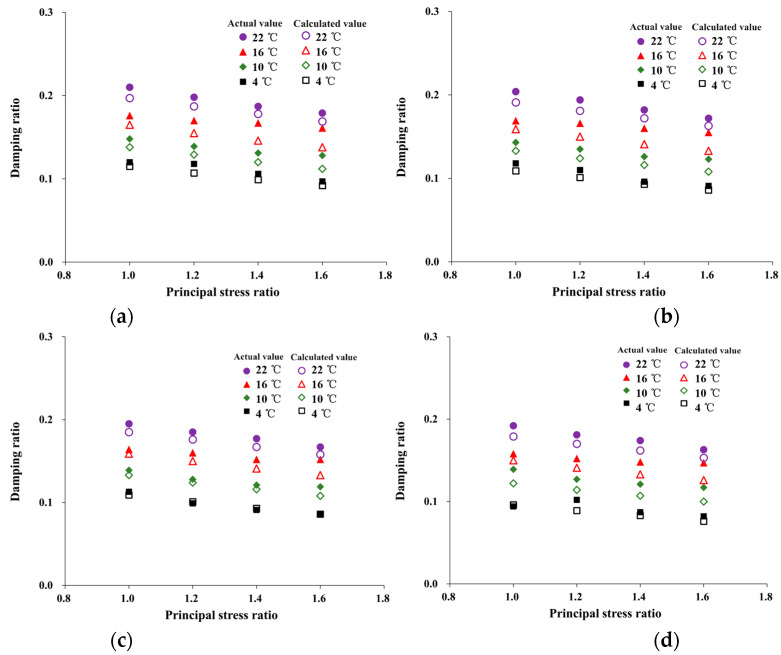
Comparison of the actual and calculated values of damping ratio modulus. (**a**) 0.5 MPa. (**b**) 0.6 MPa. (**c**) 0.7 MPa. (**d**) 0.8 MPa.

Additionally, similar to the analysis process for the maximum dynamic elastic modulus, the normalization formula for the damping ratio can be expressed as Equation (16) as follows:

(16) $$\frac{\lambda }{\left(0.2547\exp\left(-0.408T^{*}\right)\right)exp(-(0.5647T^{*}-0.439T^{*}+0.2576)K_{C})}=\left(0.3431T^{*2}-0.1562T^{*}+1.1714\right)\exp\left(-\left(0.522T^{*2}-0.2268T^{*}+0.3159\right)\sigma_{3}\right)\;\times \;F_{\sigma 3}$$

where *F_σ3_* is the confining pressure correction factor (*F_σ3_*= 0.8 at 4 °C and *σ_3_* = 0.6 or 0.7 MPa, *F_σ3_* = 0.7 at 4 °C and *σ_3_* = 0.8 MPa). Using Equation (16), the damping ratio of HAC under different conditions was calculated and compared with experimental results, as shown in Figure 17.

From the data in Figure 17, the comparison results prove that the normalization equation proposed in this paper was applicable to reflect the damping ratio of HAC under different temperatures and stress states. The relative error between actual and calculated values was within 15% and mostly within 10%.

Table 7 shows the comparison between the calculation results of Equation (16) and the damping ratio provided by [38]. The relative errors are 2.82%, 4.58%, 1.37%, and 3.60%, which are very consistent with the provided results.

## 5. Conclusions

A series of dynamic triaxial experiments of HAC were conducted under different temperatures (*T* = 4 °C, 10 °C, 16 °C, and 22 °C) and stress states (*K_c_* = 1.0, 1.2, 1.4, and 1.6; *σ_3_* = 0.5, 0.6, 0.7, and 0.8 MPa). Moreover, 300 cyclic load tests were conducted on some specimens. Based on the results, the following conclusions were drawn:

HAC exhibited a higher dynamic elastic modulus and a smaller damping ratio at low temperatures, high principal stress ratios, and high confining pressures.

HAC had good seismic and impermeable properties. There was no significant deformation of the HAC in the 300 cyclic loads test, and the permeability coefficients of the specimens after cycling all meet the specification requirements.

The logistic function better reflected the trend of the temperature influence factor of hydraulic asphalt concrete with temperature. At the same time, the damping ratio temperature sensitivity factor effectively reflected the magnitude of the sensitivity of the damping ratio to the temperature at different stress states.

Maximum dynamic elastic modulus and damping ratio calculation models for the interaction of temperature and stress states were developed using the normalization method. The model can achieve a rapid estimation of the maximum dynamic elastic modulus and damping ratio of HAC for temperatures ranging from 4 °C to 22 °C, principal stress ratios ranging from 1.0 to 1.6, and peripheral pressures ranging from 0.5 MPa to 0.8 MPa.

## Figures and Tables

**Table 1:** Properties of Karamay 70#asphalt.

Test Properties	Standard	Test Result	Test Method
Penetration (25 °C, 5 s, 100 g)/(0.1 mm)	60–80	72.8	DL-T5362-2018 [17]
Softening point/(°C)	≥45	48.2	
Ductility (5 cm/min, 15 °C)/(cm)	≥150	157	
Density (25 °C)/(g/cm^3^)	-	0.989	
**TFOT**			
Loss by mass (%)	±0.8	0.028	
Residual penetration (%)	≥61	77	
Residual ductility (5 cm/min, 15 °C)/(cm)	≥80	91	

**Table 2:** Selection of the levels of factors.

Factors	The Level of Factor
Bitumen aggregate ratio	6.5%, 7.5%, 8.5%
Filler content	11%, 13%, 15%
Graded index	0.36, 0.39, 0.42

**Table 3:** Asphalt mixture proportion.

Particle Size (mm)	Coarse Aggregate					Fine Aggregate					Fine
	19.00–16.00	16.00–13.20	13.20–9.50	9.50–4.75	4.75–2.36	2.36–1.18	1.18–0.60	0.60–0.30	0.30–0.15	0.15–0.075	<0.075
Percent (%)	6.52	6.80	10.51	18.19	13.99	10.57	7.89	6.19	4.73	3.61	11.00

**Table 4:** *a*, *b*, *k*, and *R^2^* under different stress states.

*σ_3_*/MPa	*K_c_*	*k*	*a*	*b*	*R^2^*
0.5	1.0	2.211	6.978	4.914	0.999
	1.2	2.346	3.411	4.190	0.999
	1.4	2.484	2.808	3.967	0.998
	1.6	2.601	2.302	3.962	0.998
0.6	1.0	2.425	3.536	4.113	0.999
	1.2	3.048	2.910	2.743	0.999
	1.4	3.135	2.098	2.504	0.993
	1.6	3.094	1.816	2.843	0.999
0.7	1.0	2.599	1.885	3.992	0.990
	1.2	3.223	1.392	2.209	0.998
	1.4	3.029	1.205	3.087	0.998
	1.6	3.615	1.047	1.582	0.985
0.8	1.0	2.888	1.199	3.305	0.999
	1.2	3.304	0.922	1.961	0.999
	1.4	3.291	0.805	2.529	0.989
	1.6	3.953	1.041	1.596	0.978

**Table 5:** *B_λ_* and *R^2^* under different stress states.

*σ_3_*/MPa	*K_c_*	*B_λ_*	*R^2^*
0.5	1.0	3.344	0.998
	1.2	3.439	0.998
	1.4	3.560	0.999
	1.6	3.652	0.999
0.6	1.0	3.410	0.997
	1.2	3.512	0.998
	1.4	3.657	0.999
	1.6	3.735	0.999
0.7	1.0	3.488	0.998
	1.2	3.656	0.998
	1.4	3.750	0.999
	1.6	3.846	0.999
0.8	1.0	3.632	0.998
	1.2	3.656	0.998
	1.4	3.750	0.999
	1.6	3.846	0.999

**Table 6:** Comparison between calculated and provided values of maximum dynamic elastic madulus.

Various Factors and Levels			Maximum Dynamic Elastic Modulus		
*T* (°C)	*K_c_* -	*σ_3_* (MPa)	Provided Value /MPa	Calculated Value /MPa	Relative Error /%
9	1.5	0.6	1200	996.35	16.97
		0.8	1700	1957.26	15.13
20	1.2	0.5	730	666.06	8.76
	1.5	0.5	730	771.75	5.71

**Table 7:** Comparison between calculated and provided values of damping ratio.

Various Factors and Levels			Damping Ratio		
*T* (°C)	*K_c_* -	*σ_3_* (MPa)	Provided Value	Calculated Value	Relative Error /%
5.4	1.2	0.6	0.054	0.055	2.82
	1.5		0.049	0.051	4.58
15.4	1.2	0.6	0.073	0.074	1.37
	1.5		0.060	0.057	3.60

## Data Availability

The data used to support the findings of this study are available from the corresponding author upon reasonable request.
